# Explainable Artificial Intelligence (XAI) for 3D Medical Imaging: A Scoping Review of Volumetric Attribution Methods

**DOI:** 10.3390/jimaging12100498

**Published:** 2026-10-09

**Authors:** Deepshikha Bhati, Mrinaal Rajesh Nahata, Fnu Neha, Ruthra Bellan, Deepali Bhati

**Affiliations:** 1 Department of Computer Science, Kent State University, Kent, OH 44242, USA; mnahata@kent.edu (M.R.N.); neha@kent.edu (F.N.); 2 Department of Computer Science, SRH University of Applied Sciences, 12059 Berlin, Germany; ruthra.bellan@srh-hochschulen.de; 3 International Education College, Xinjiang Medical University, Urumqi 830091, China; deepalibhati99@gmail.com

**Keywords:** explainable artificial intelligence (XAI), deep learning, Grad-CAM, Layer-wise Relevance Propagation (LRP), Integrated Gradients, SHAP, volumetric visualization, 3D medical imaging, CT, MRI, PET, healthcare

## Abstract

Explainable artificial intelligence (XAI) methods developed for two-dimensional images may not preserve spatial context when applied to volumetric medical imaging. This scoping review map attribution methods for 3D medical images and examines how dimensionality is preserved across model inference, attribution generation, and visualization. Following PRISMA-ScR guidance, we searched IEEE Xplore, PubMed, ACM Digital Library, Semantic Scholar, and arXiv for studies published from January 2018 to May 2026. We mapped 32 selected studies by attribution method, imaging modality, clinical task, evaluation approach, and dimensionality paradigm. Grad-CAM and its variants were the most frequently reported method family. Within the selected corpus, two studies were classified as P2 boundary cases, 29 as P3, and one as P4. Most studies using volumetric inference presented explanations through two-dimensional views, and quantitative evaluation of attribution quality was uncommon. These findings identify a gap between volumetric model inference and explanation presentation and motivate improved volumetric visualization and more consistent evaluation.

## 1. Introduction

Medical imaging is a cornerstone of modern healthcare, providing critical insights for diagnosing, treating, and monitoring a wide range of diseases [1]. Major clinical modalities, including computed tomography (CT), magnetic resonance imaging (MRI), positron emission tomography (PET), single-photon emission computed tomography (SPECT), and three-dimensional optical coherence tomography (3D OCT), acquire volumetric data that preserve anatomical structures and spatial relationships across three dimensions.

The growing adoption of deep learning (DL) has further expanded the capabilities of medical image analysis, with models achieving strong performance in classification, detection, segmentation, prognosis, and treatment planning [2]. As these models become increasingly integrated into clinical decision-support systems, understanding the basis of their predictions has become as important as achieving high predictive accuracy. The complex and opaque nature of modern DL architectures raises concerns regarding transparency, reliability, and clinical trustworthiness in safety-critical healthcare environments [3].

Explainable artificial intelligence (XAI) seeks to address these concerns by identifying the input features or image regions that contribute to a model prediction. Representative post-hoc attribution methods include Gradient-weighted Class Activation Mapping (Grad-CAM) [4], Layer-wise Relevance Propagation (LRP) [5], Integrated Gradients (IG) [6], SHapley Additive exPlanations (SHAP) [7], and Local Interpretable Model-agnostic Explanations (LIME) [8]. These methods typically assign attribution, saliency, or relevance scores to image regions and present the resulting explanations as heatmaps.

Despite this progress, most XAI attribution methods were originally developed for two-dimensional images [2,9]. In volumetric medical imaging, explanations are often generated slice by slice or presented through selected axial, coronal, or sagittal views, even when the underlying model processes a complete 3D volume. This creates a dimensionality gap between the way a model analyzes volumetric information and the way its prediction is explained. As a result, inter-slice continuity, lesion extent, and spatial relationships across anatomical structures cannot be fully represented in the final explanation.

Applying attribution methods to volumetric images introduces challenges at several stages. A 3D scan contains substantially more voxels than a 2D image, increasing the computational cost of gradient integration, perturbation, and Shapley-value estimation. Downsampling within a 3D network can produce coarse attribution maps that obscure small lesions even when the model uses the complete volume. Slice-based explanations may omit inter-slice continuity, while displaying a volumetric attribution map only as selected slices can make the spatial extent of a finding difficult to inspect. These issues also complicate evaluation: an anatomical mask may indicate a clinically relevant region without establishing that the model actually relied on that region for its prediction.

The way a volumetric attribution is visualized has direct implications for its interpretation. Slice overlays are familiar to radiologists and permit detailed inspection within an axial, coronal, or sagittal plane; selected slices may omit attributed regions and make it difficult to assess lesion extent, inter-slice continuity, and relationships between spatially separated structures. Volumetric renderings, isosurfaces, and interactive views can preserve these relationships, but their interpretation may depend on threshold selection, opacity settings, viewpoint, and the occlusion of internal structures. Accordingly, native 3D visualization should complement rather than replace clinically familiar slice-based inspection. Respond-CAM, for example, demonstrated projected three-dimensional contours and isosurfaces together with corresponding two-dimensional slices [9], whereas most subsequent clinical studies continued to present volumetric attributions primarily through selected slice overlays.

Quantitative assessment is equally important because visual plausibility does not establish that an explanation faithfully represents a model’s reasoning. Localization metrics evaluate agreement with lesion or anatomical masks, faithfulness metrics measure how predictions change when attributed regions are perturbed, and robustness measures examine the stability of explanations under small input or model changes. Recent studies illustrate these complementary perspectives. SE3D introduced volumetric localization measures including VxAP, MaxF1, and Max3DBoxAcc [10]; Chakraborty et al. [11] and Chrabaszcz et al. [12] employed perturbation-based measures such as AOPC and AUPC to evaluate faithfulness. Nevertheless, localization against an anatomical mask does not necessarily demonstrate that the model relied on that region, while a faithful attribution is not automatically clinically meaningful. Volumetric XAI requires complementary evaluation across localization, faithfulness, robustness, and clinical-expert assessment.

**Figure 1:**
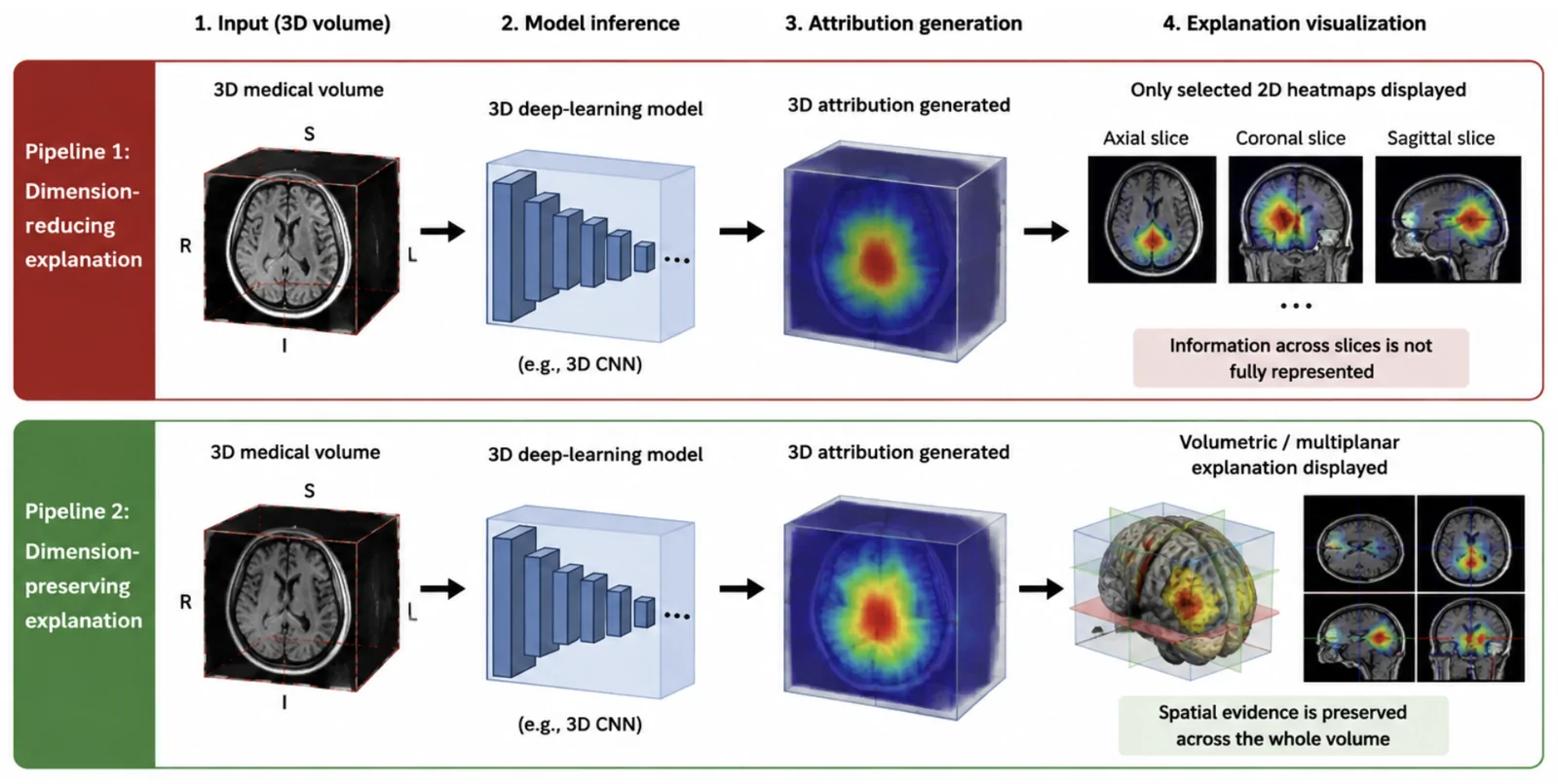
Illustration of the dimensionality gap in explainable 3D medical imaging. In a dimension-reducing pipeline, a volumetric scan may be processed by a 3D model, while the resulting attribution is presented only through selected 2D slices. In a dimension-preserving pipeline, volumetric information is retained during model inference, attribution generation, and explanation visualization.

This distinction is important because volumetric explainability involves more than the use of a 3D model. Dimensionality can be altered at several stages, including input preparation, model inference, attribution generation, and explanation visualization. A model processes a complete 3D scan while the resulting attribution is shown only as individual 2D overlays. Conversely, a slice-based model generates multiple 2D explanations that are later combined into an apparent volumetric representation. These pipelines (see Figure 1) differ substantially in the extent to which three-dimensional information is preserved.

For example, when a DL model predicts Alzheimer’s disease from an entire brain MRI volume, its decision depends on distributed anatomical patterns across multiple regions and slices. Presenting the explanation only through isolated 2D views makes these relationships difficult to interpret. Similar concerns arise in brain tumor analysis, lung nodule assessment, prostate imaging, and other applications in which disease characteristics depend on 3D location, morphology, and extent.

Previous reviews have examined XAI in medical imaging from the perspectives of method categories, clinical applications, and interpretability challenges [2]. Limited attention has been given to how dimensionality is preserved across the complete explanation pipeline. The literature also lacks a systematic comparison of attribution methods in terms of imaging modality, clinical task, evaluation strategy, and volumetric visualization capability. A focused synthesis is needed to identify current practices, methodological limitations, and emerging directions in explainable 3D medical imaging.

The objective of this review is to map attribution approaches used with volumetric medical examinations and determine where three-dimensional information is preserved or reduced across model inference, attribution, and visualization.

To address this need, this review is guided by the following research questions (RQs):

RQ1: What XAI attribution methods have been applied to 3D medical imaging, and how have they evolved between 2018 and 2026?
RQ2: What imaging modalities, clinical tasks, anatomical regions, and disease targets have been studied in the existing literature?
RQ3: To what extent do current approaches generate genuinely volumetric explanations, and what evaluation frameworks have been used to assess them?
RQ4: What limitations, research gaps, and future research opportunities remain for XAI in 3D medical imaging?

Based on these research questions, this review makes the following contributions:

- It presents a PRISMA-ScR-guided scoping review of XAI attribution methods for 3D medical imaging published between 2018 and 2026.
- It introduces a four-paradigm dimensionality taxonomy that characterizes the progression from slice-based explanations to fully volumetric attribution and visualization.
- It establishes consistent mathematical notation for representative attribution methods, including Grad-CAM, LRP, IGs, and SHAP.
- It compares the reviewed studies across imaging modalities, clinical applications, evaluation strategies, and explanation dimensionality.
- It identifies major research gaps and outlines future directions toward resolution-preserving attribution, segmentation-specific explainability, standardized benchmarking, and clinical translation.

The remainder of this paper is organized as follows: Section 2 describes the systematic review methodology. Section 3 presents the mathematical background and introduces the proposed dimensionality taxonomy. Section 4 reports the results of the literature review. Section 5 discusses the findings and their implications. Section 6 outlines the major open challenges and future research directions. Finally, Section 7 concludes the paper.

## 2. Research Methodology

This study follows a systematic scoping review methodology and is reported in accordance with the Preferred Reporting Items for Systematic Reviews and Meta-Analyses Extension for Scoping Reviews (PRISMA-ScR) guidelines [13]. A scoping review design was selected because the objective was to map the range of XAI attribution methods applied to 3D medical imaging, characterize their methodological and clinical applications, and identify gaps in explanation dimensionality and evaluation practices. The review protocol was not prospectively registered, and a formal written protocol was not prepared or made publicly available.

The review process consisted of four stages: literature identification, study screening, data extraction, and qualitative synthesis. Before the search was conducted, the research questions, eligibility criteria, extraction variables, and dimensionality categories were defined to improve consistency throughout the review process.

### 2.1. Search Strategy

A systematic literature search was conducted across IEEE Xplore, PubMed, ACM Digital Library, Semantic Scholar, and arXiv. The arXiv search was limited to the cs.CV, cs.LG, and eess.IV categories. These sources were selected to capture peer-reviewed and preprint literature spanning medical imaging, computer vision, machine learning, and XAI.

IEEE Xplore and ACM Digital Library were included to capture engineering and computing research; PubMed was included for biomedical and clinical imaging research; Semantic Scholar was used for broader interdisciplinary discovery; and arXiv was included to identify relevant preprints in the specified computer-vision, machine-learning, and imaging categories. The search was limited to these sources, so studies indexed only in other databases may have been missed.

The search covered studies published between January 2018 and May 2026. The final literature search was conducted in May 2026. The primary search combined terms related to explainability, volumetric analysis, and medical imaging. The following expression summarizes the search concepts; it is not a verbatim query executed identically across all five sources:

(explainable AI” OR XAI” OR explainability” OR saliency” OR attribution”)

AND (3D” OR volumetric” OR three-dimensional”) AND (medical imaging” OR MRI” OR CT” OR PET” OR SPECT” OR OCT”)

The query was adapted to the syntax and indexing structure of each database. Additional searches combined the names of individual attribution methods, such as Grad-CAM, LRP, IGs, SHAP, and LIME, with specific imaging modalities. Forward and backward citation searching was also performed for influential and highly relevant studies to improve retrieval coverage.

### 2.2. Study Selection

Studies were screened according to predefined inclusion and exclusion criteria. Two reviewers independently screened the titles and abstracts of the retrieved records. Potentially eligible studies were subsequently assessed through full-text review against the predefined inclusion and exclusion criteria. Disagreements regarding study eligibility were resolved through discussion until consensus was reached.

Inclusion criteria: Eligible studies were required to (1) apply or evaluate an XAI attribution method, (2) analyze inherently volumetric medical imaging data, including CT, MRI, PET, SPECT, or 3D OCT, (3) generate voxel-level or region-level spatial attribution maps as a primary or secondary output, and (4) be published in English as peer-reviewed articles or publicly available preprints. One study that used class activation maps for training rather than as a standalone explanation output was retained solely as a contextual P2 boundary case; it is identified as such in Table 1.

Exclusion criteria: Studies were excluded if they (1) analyzed only native 2D images, with no volumetric medical examination as the source; (2) provided only scalar or textual explanations without spatial attribution; (3) employed attention mechanisms solely to improve model performance without generating explainable attribution maps, except for the explicitly identified P2 boundary case; or (4) addressed application domains outside medical imaging.

Operational definition of volumetric model inference: A model was classified as using 3D inference when its prediction network jointly processed a volume or volumetric patch with an explicit depth dimension. This definition includes 3D CNNs, 3D ResNets, 3D DenseNets, 3D U-Nets, and volumetric transformer or hybrid architectures when their inference incorporates spatial information across slices. A model applying a 2D network independently to individual slices was classified as P2, even if its predictions or images were subsequently stacked into a 3D reconstruction. A multi-slice or “2.5D” model was classified according to whether its inference jointly modeled the depth dimension; the number of input slices or use of a “3D” label alone was not decisive.

Operational definition of spatial attribution: We recorded whether an explanation assigned importance to individual voxels, voxel groups, patches, supervoxels, or anatomical regions. These were distinguished in data extraction rather than all described as voxel-wise attribution. Eligible spatial approaches could include 3D CAM/Grad-CAM, gradients, Integrated Gradients, LRP, SHAP, occlusion, and other perturbation methods when they produced a spatial contribution map for a model output. Attention maps or feature visualizations were counted as explanations only when the study explicitly used them to explain an output; their presence inside a model alone was insufficient.

The 32-study corpus includes 30 studies using volumetric model inference (P3–P4) and two deliberately retained P2 studies that process slices from volumetric examinations independently. The P2 studies serve as boundary cases for the dimensionality taxonomy: they show information loss before model inference, whereas P3 studies show information loss at the visualization stage. The two P2 studies are analyzed separately and are not claimed to constitute an exhaustive sample of slice-based XAI research.

### 2.3. Data Extraction and Analysis

A standardized data extraction protocol was used to collect information from each eligible study, including publication year, venue, imaging modality, anatomical region, clinical task, disease target, attribution method, deep learning architecture, dataset, explanation dimensionality, evaluation methodology, and reported limitations.

To improve consistency, two reviewers independently categorized each study according to the proposed dimensionality taxonomy. Inter-rater agreement was evaluated using Cohen’s κ, yielding a value of 0.83, indicating substantial agreement according to the Landis–Koch interpretation scale [42]. Any disagreements were resolved through discussion until consensus was reached.

Consistent with PRISMA-ScR guidance for scoping reviews, a formal risk-of-bias or critical-appraisal assessment of individual studies was not performed. This is appropriate for a scoping review, whose objective is to map the extent, range, and nature of existing XAI attribution methods for 3D medical imaging rather than to evaluate intervention effectiveness or pool outcome estimates. Instead, methodological rigor and reporting quality were considered qualitatively during data extraction, and are reflected in the reported evaluation strategies (Section 4.5) and study limitations (Table 1).

As this review does not estimate pooled effect sizes for a specific clinical outcome, a formal certainty-of-evidence assessment (e.g., GRADE) was not applicable and was not performed.

A formal assessment of reporting bias, such as funnel plot asymmetry or Egger’s test, was not conducted, as these methods are designed for meta-analyses of pooled effect estimates and are not applicable to a qualitative, methods-mapping scoping review. The search strategy was limited to four bibliographic search platforms and arXiv in the cs.CV, cs.LG, and eess.IV categories; grey literature, unpublished results, and preprint servers outside arXiv were not systematically searched. Consequently, studies with null or negative findings on XAI attribution performance may be underrepresented in the reviewed literature, and this possibility is noted as a limitation of the review process (Section 5.6).

The extracted data were synthesized qualitatively to analyze publication trends, attribution methods, application domains, evaluation strategies, and the progression toward genuinely volumetric explanations. To explore possible sources of variability among study results, studies were qualitatively compared across attribution method family, imaging modality, clinical task, and dimensionality paradigm (P2–P4), rather than through formal subgroup analysis or meta-regression, consistent with the narrative synthesis approach of a scoping review.

### 2.4. PRISMA Screening Process

**Figure 2:**
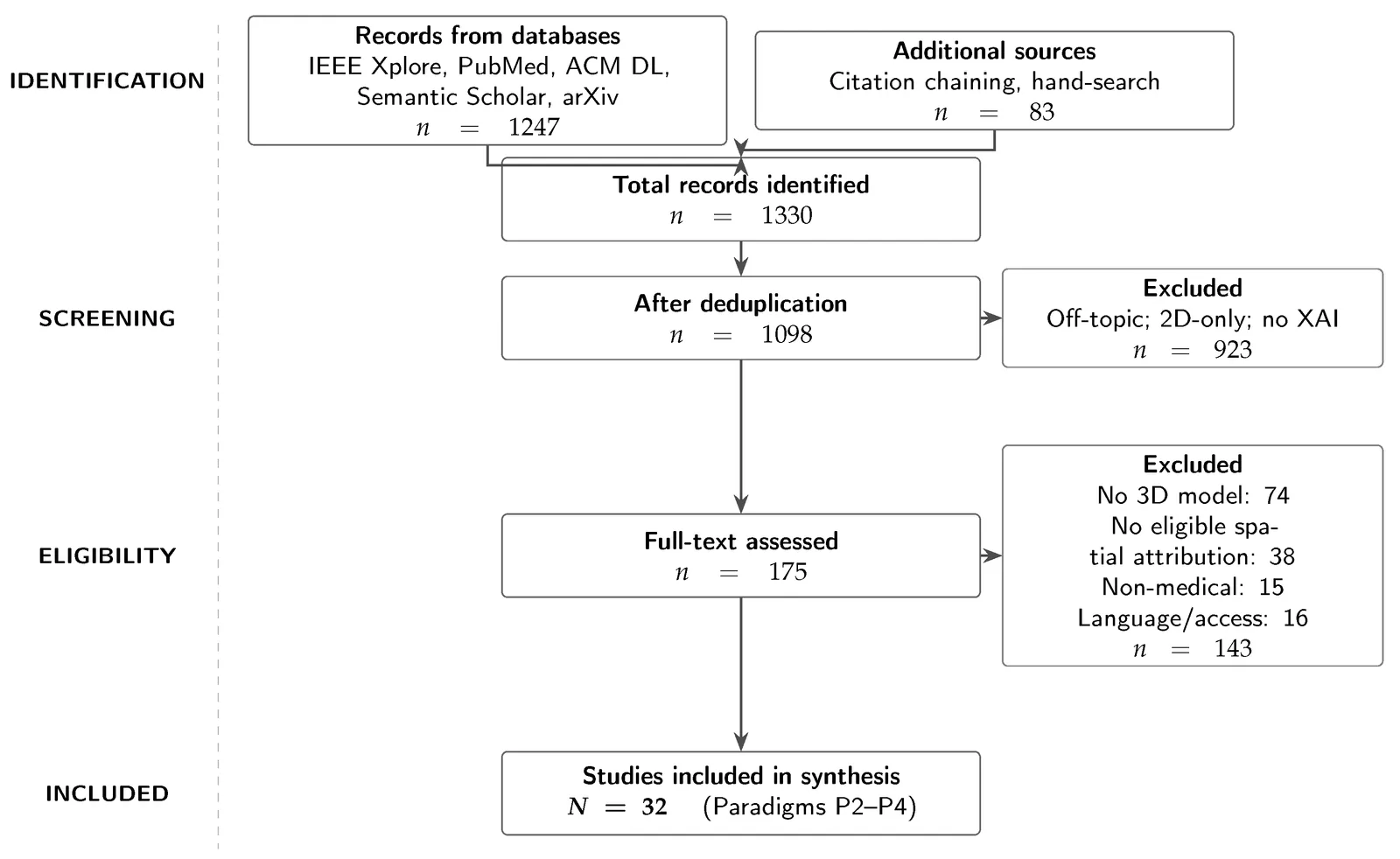
PRISMA-ScR study-selection flow. Database searching identified 1247 records, and citation chaining and manual searching identified 83 additional records. After 232 duplicates were removed, 1098 titles and abstracts were screened and 175 reports underwent full-text assessment. A total of 143 reports were excluded for the reasons shown, leaving 32 studies in the qualitative synthesis. The final corpus includes 30 studies using volumetric model inference and two explicitly identified P2 boundary cases using independent slices from volumetric examinations.

Figure 2 summarizes the PRISMA-ScR study selection process. Records identified through database searching and additional sources underwent duplicate removal, title and abstract screening, full-text eligibility assessment, and final inclusion based on the predefined selection criteria. Reasons for exclusion at the full-text stage were recorded and are reported in the PRISMA-ScR flow diagram. The resulting corpus forms the basis for the comparative analyses and taxonomy presented in this review.

Given the large number of full-text exclusions (n=143), reasons for exclusion are summarized by category in Figure 2. This aggregate reporting provides a transparent overview of the primary reasons for exclusion during the full-text eligibility assessment.

The relatively small final corpus reflects the narrow intersection required by the review scope. A study generally had to examine an inherently volumetric medical examination, apply or evaluate a spatial explanation approach, and provide sufficient information to determine the dimensionality of model inference, attribution, and visualization. Studies outside medical imaging, studies without spatial attribution, and studies without eligible volumetric model inference were excluded. The two explicitly identified P2 studies were retained as contextual boundary cases for the proposed taxonomy.

## 3. Background and Mathematical Foundations

This section presents the mathematical foundations of representative XAI attribution methods for volumetric medical imaging. It establishes consistent notation for comparing these methods, followed by commonly used evaluation metrics and the proposed dimensionality taxonomy.

### 3.1. Unified Notation for Volumetric Attribution

Let $f:X\to R^{C}$ denote a deep neural network that maps a volumetric medical image $x\in R^{D\times H\times W}$ to a vector of class logits, where *D*, *H*, and *W* represent the depth, height, and width of the input volume, respectively. For a target class *c*, the corresponding prediction score is

$$S_{c}\left(x\right)={\left[f\left(x\right)\right]}_{c}.$$

An attribution method produces a voxel-wise relevance map

(1) $$A_{c}:R^{D\times H\times W}⟶R^{D\times H\times W},$$

where each voxel (d,h,w) is assigned a relevance value $r_{d,h,w}\in R$ that quantifies its contribution to the prediction for class *c*.

### 3.2. Gradient-Based Saliency

Gradient-based attribution methods estimate voxel importance by measuring the sensitivity of the target score to changes in the input volume [43]. The attribution map is defined as

(2) $$A_{c}^{\mathrm{grad}}\left(x\right)\;=\;\left|\;\frac{\partial S_{c}\left(x\right)}{\partial x}\;\right|.$$

Large gradient magnitudes indicate voxels for which small intensity changes strongly affect the target prediction.

### 3.3. 3D Gradient-Weighted Class Activation Mapping (Grad-CAM)

Unlike input-gradient methods, Gradient-weighted Class Activation Mapping (Grad-CAM) generates class-discriminative explanations from intermediate convolutional feature maps. Originally proposed for 2D convolutional neural networks (CNNs) [4], Grad-CAM has been extended to volumetric networks and remains the most widely adopted attribution technique in 3D medical imaging. Let $A^{k}\in R^{d^{\prime }\times h^{\prime }\times w^{\prime }}$ denote the *k*-th activation map at the final convolutional layer, where $d^{\prime }\leq D$, $h^{\prime }\leq H$, $w^{\prime }\leq W$. The class-discriminative weight is:

(3) $$\alpha_{k}^{c}\;=\;\frac{1}{d^{\prime }\;h^{\prime }\;w^{\prime }}\sum_{i=1}^{d^{\prime }}\sum_{j=1}^{h^{\prime }}\sum_{l=1}^{w^{\prime }}\frac{\partial S_{c}}{\partial A_{i,j,l}^{k}},$$

and the 3D Grad-CAM map, after trilinear upsampling ↑ to input resolution, is:

(4) $$L_{c}^{3D\text{-}\mathrm{GradCAM}}\;=\;\mathrm{ReLU}\;\left(↑\;\left[\;\sum_{k}\alpha_{k}^{c}\;A^{k}\right]\right).$$

The ReLU operator retains only features that contribute positively to the target-class score. to the target class prediction. A fundamental limitation of Grad-CAM arises from the progressive reduction in spatial resolution introduced by pooling operations within deep convolutional networks.

(5) $$d^{\prime }=\frac{D}{2^{P}},\;h^{\prime }=\frac{H}{2^{P}},\;w^{\prime }=\frac{W}{2^{P}}.$$

For a $128^{3}$ input volume with four pooling stages, the final activation map has a spatial resolution of only $8^{3}$. After trilinear upsampling, each attribution voxel represents a $16^{3}$ (4096-voxel) region, corresponding to approximately $4.1\;{\mathrm{cm}}^{3}$ for MRI volumes with $1\;{\mathrm{mm}}^{3}$ isotropic resolution. Such coarse localization may obscure small pathological structures, including multiple sclerosis plaques and other focal lesions, highlighting one of the principal limitations of Grad-CAM for volumetric medical imaging.

### 3.4. Integrated Gradients

Integrated Gradients (IG) addresses the gradient saturation problem by accumulating gradients along a continuous path from a reference baseline to the input volume [6]. Unlike simple gradient saliency, IG satisfies desirable axiomatic properties, including implementation invariance and completeness.

(6) $$A_{c}^{\mathrm{IG}}\left(x\right)\;=\;\left(x-\bar{x}\right)\;⊙\;\int_{0}^{1}\frac{\partial S_{c}\;\left(\bar{x}+\alpha \left(x-\bar{x}\right)\right)}{\partial x}\;d\alpha ,$$

where ⊙ denotes the Hadamard product. IG satisfies the *completeness* axiom:

(7) $$\sum_{d,h,w}{A_{c}^{\mathrm{IG}}\left(x\right)}_{d,h,w}\;=\;S_{c}\left(x\right)-S_{c}\left(\bar{x}\right),$$

### 3.5. Layer-Wise Relevance Propagation

Layer-wise Relevance Propagation (LRP) explains predictions by propagating the output score backward through the network and redistributing prediction evidence according to relevance-propagation rules [5].

(8) $$R_{j}^{\left(l\right)}\;=\;\sum_{k}\frac{a_{j}^{\left(l\right)}\;w_{jk}}{\sum_{j^{\prime }}a_{j^{\prime }}^{\left(l\right)}w_{j^{\prime }k}+\varepsilon }\;R_{k}^{\left(l+1\right)},$$

where $R_{j}^{\left(l\right)}$ is the relevance of neuron *j* in layer *l*, $a_{j}^{\left(l\right)}$ is its activation, $w_{jk}$ is the weight connecting neuron *j* to neuron *k*, and ε>0 is a numerical stabilizer.

For propagation rules designed to conserve relevance, the relevance received by one layer is redistributed to the preceding layer. For the ε-stabilized rule in Equation (8), conservation is generally approximate because the stabilizing term may absorb a small amount of relevance:

(9) $$\sum_{j}R_{j}^{\left(l\right)}\;\approx \;\sum_{k}R_{k}^{\left(l+1\right)}.$$

Exact equality requires a strictly conservation-preserving propagation rule and appropriate treatment of bias and stabilization terms. At the input layer, the resulting relevance values provide a voxel-level decomposition of the selected model output. LRP can propagate relevance back to the input dimensions without relying on upsampling a final convolutional feature map, the resulting map should not automatically be interpreted as having perfect spatial precision.

### 3.6. SHAP for Volumetric Inputs

SHAP interprets model predictions using Shapley values from cooperative game theory [7]. In volumetric imaging, a feature may represent an individual voxel or a predefined group of voxels, such as a patch, supervoxel, or anatomical region. Let Ω denote the set of attribution units. For an attribution unit i∈Ω, its Shapley value is

(10) $$\phi_{i}\;=\;\sum_{S\subseteq \Omega \setminus \{i\}}\frac{|S|!\;(|\Omega |-|S|-1)!}{|\Omega |!}\left[v(S\cup \{i\})-v(S)\right],$$

Exact Shapley-value computation requires enumeration over an exponential number of feature coalitions and is intractable for high-dimensional medical volumes. Practical implementations use approximations, background distributions, or grouped features. DeepSHAP combines DeepLIFT-style backpropagation with reference samples to approximate SHAP values; its computational requirements depend on the network, the selected background samples, and the definition of the attribution units.

### 3.7. Evaluation Metrics for 3D Attribution Quality

Attribution quality is commonly evaluated using two complementary criteria: *faithfulness* and *localization*. Faithfulness assesses whether highly attributed voxels influence the model prediction. Removing the most relevant features first (MoRF) should substantially reduce prediction confidence, whereas removing the least relevant features first (LeRF) should have minimal effect.

Area Over the Perturbation Curve for Correctness (AOPC) quantifies faithfulness [44]:

(11) $${\mathrm{AOPC}}_{\mathrm{corr}}\;=\;\frac{1}{K}\sum_{k=1}^{K}\left[S_{c}\left(x\right)-S_{c}\;\left(x_{\mathrm{MoRF}}^{\left(k\right)}\right)\right],$$

where $x_{\mathrm{MoRF}}^{\left(k\right)}$ denotes the perturbed input obtained by replacing the top-*k* ranked voxels with a baseline value. Larger AOPC values indicate greater faithfulness.

Localization measures agreement between an attribution map and a binary lesion or anatomical mask $G\in {\left\{0,1\right\}}^{D\times H\times W}$. Voxel Average Precision (VxAP), used in SE3D [10], extends pixel average precision to volumetric attribution. For an attribution threshold τ, voxel-level precision and recall are defined as

(12) $$\text{Prec}\left(\tau \right)=\frac{\left|\left\{\;v:A_{c}{\left(x\right)}_{v}\geq \tau \;\right\}\cap \left\{\;v:G_{v}=1\;\right\}\right|}{\left|\{\;v:A_{c}{\left(x\right)}_{v}\geq \tau \;\}\right|},$$

(13) $$\text{Rec}\left(\tau \right)=\frac{\left|\left\{\;v:A_{c}{\left(x\right)}_{v}\geq \tau \;\right\}\cap \left\{\;v:G_{v}=1\;\right\}\right|}{\left|\{\;v:G_{v}=1\;\}\right|}.$$

For decreasing thresholds $\tau_{1},\ldots ,\tau_{L}$, VxAP can be written as

(14) $$\mathrm{VxAP}=\sum_{\ell =1}^{L}\left[\text{Rec}\left(\tau_{\ell }\right)-\text{Rec}\left(\tau_{\ell -1}\right)\right]\text{Prec}\left(\tau_{\ell }\right).$$

Higher VxAP values indicate stronger voxel-level agreement between the attribution ranking and the reference mask. Such agreement measures localization rather than model faithfulness.

### 3.8. Dimensionality Paradigm Taxonomy

**Figure 3:**
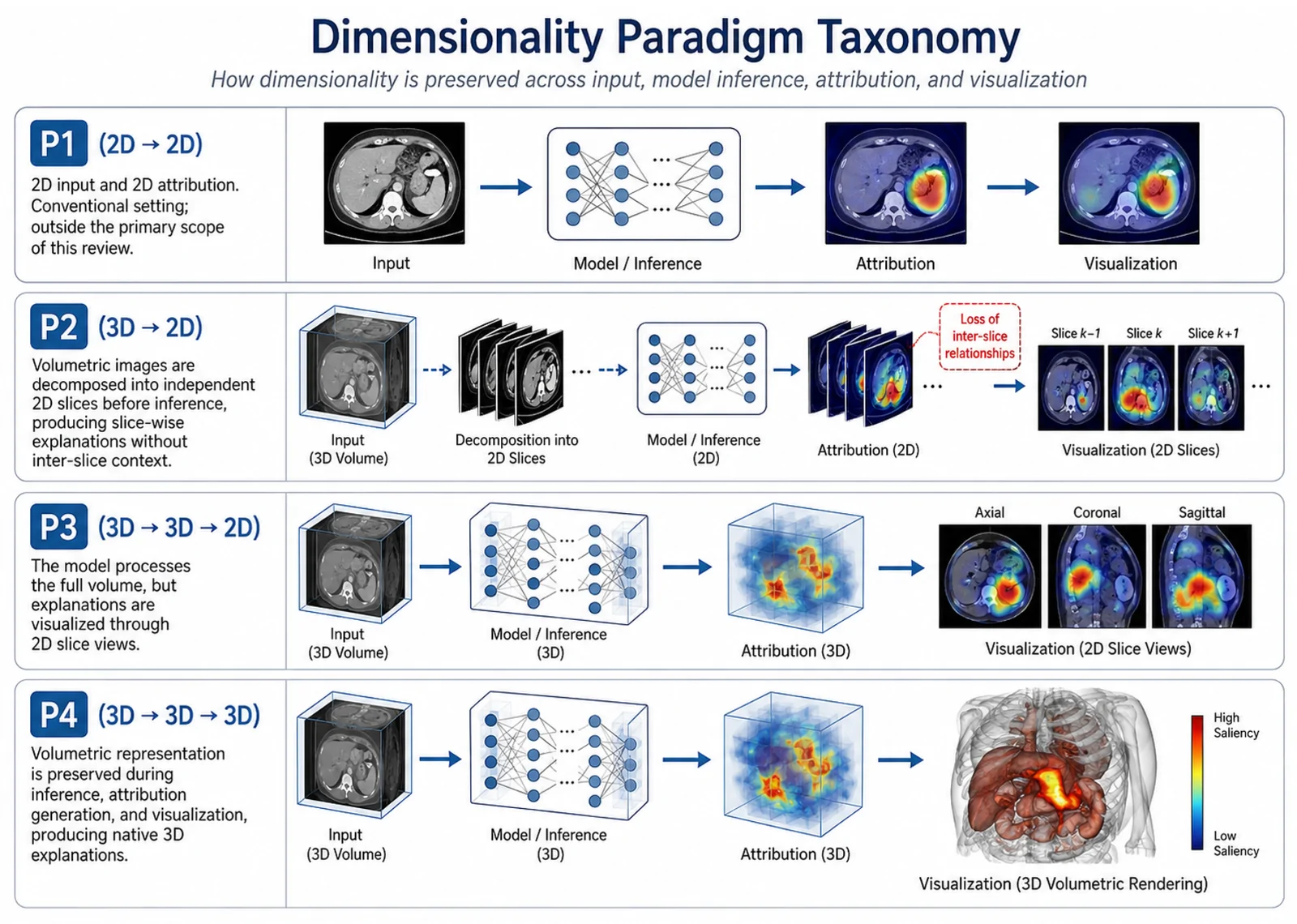
Proposed dimensionality paradigm taxonomy for XAI in medical imaging. P1 represents the conventional 2D setting, in which both the input and attribution map are two-dimensional. In P2, a volumetric image is decomposed into independent 2D slices before inference, resulting in slice-wise explanations that do not preserve inter-slice relationships. In P3, the complete volume is processed and attributed in three dimensions, but the explanation is visualized using axial, coronal, or sagittal 2D views. P4 preserves the volumetric representation throughout model inference, attribution generation, and visualization, producing a native 3D explanation. Ellipses indicate omitted intermediate neural-network layers or additional image slices for schematic simplicity.

Existing XAI approaches differ in how dimensionality is preserved across data processing, model inference, attribution generation, and visualization. To characterize these differences, this review introduces a four-paradigm dimensionality taxonomy (P1–P4) as shown in Figure 3.

**P1 (2D → 2D):** Both the input and attribution map are two-dimensional. This conventional setting lies outside the primary scope of this review.

**P2 (3D → 2D):** Volumetric images are decomposed into independent 2D slices before model inference, producing slice-wise explanations that do not preserve inter-slice relationships.

**P3 (3D → 3D → 2D):** The model processes the complete volume, but the resulting explanation is presented through 2D slice views.

**P4 (3D → 3D → 3D):** The volumetric representation is preserved during model inference, attribution generation, and visualization, producing native 3D explanations.

Paradigm assignment was based on three recorded features: (1) whether inference jointly used inter-slice information; (2) whether the attribution was computed for a volumetric model output; and (3) whether the explanation was presented in a spatial 3D view or only as 2D slices. Independent slice inference was coded P2. Joint volumetric inference with explanation presented only as 2D views was coded P3. Joint volumetric inference with an explicitly presented 3D explanation was coded P4. Post-hoc stacking of 2D predictions or explanations did not, by itself, qualify a study as P4.

For classification purposes, displaying multiple orthogonal slices or arranging slices in a montage was treated as 2D presentation. A study was assigned to P4 only when the explanation itself was presented as a spatial 3D object, such as a volume rendering or an isosurface that preserved depth relationships. Architecture names and author claims that a method was “3D” were not sufficient for P4 assignment.

## 4. Literature Analysis

This section analyzes the included studies in terms of publication trends, imaging modalities, clinical applications, attribution methods, dimensionality paradigms, and evaluation strategies. The findings are consolidated in a comparative overview of the reviewed literature.

### 4.1. Temporal Trends in Method Adoption

**Figure 4:**
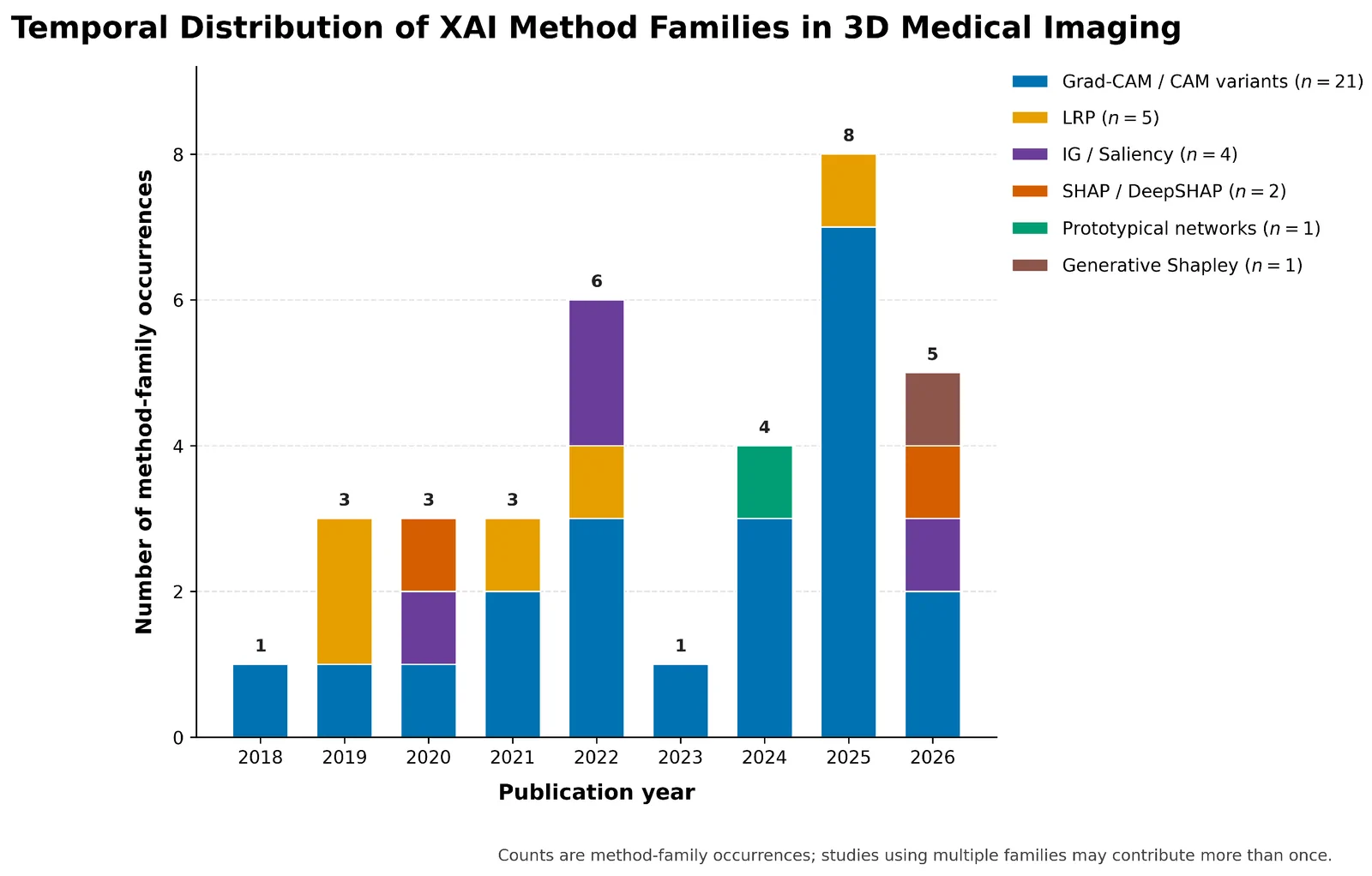
Temporal distribution of XAI method-family occurrences across the 32 included studies. Counts were derived from study-level coding of the methods applied or evaluated in each publication. A study may contribute to more than one method family; consequently, the total number of method-family occurrences exceeds the number of unique studies. Generative Shapley is represented both within the broader SHAP family and as a distinct generative explanation approach.

Figure 4 summarizes the annual occurrence of the six method families identified in the selected studies. Because studies applying or evaluating multiple method families contribute to each applicable category, the figure shows method-family occurrences rather than unique-study counts. The distribution indicates a progression from early volumetric activation mapping and relevance propagation to broader clinical adoption and, more recently, prototype-based and generative explanation approaches.

**Phase I (2018–2020): Methodological foundations.** Early studies established the feasibility of attribution methods for volumetric models. Respond-CAM extended class activation mapping to three-dimensional convolutional feature maps and demonstrated the approach using cellular electron cryo-tomography subtomograms [9], while LRP was applied to neurological disease classification [14,15]. M3D-CAM further supported adoption by providing a software framework for generating 3D activation maps [17].

Only a limited number of studies were published, they established the methodological foundations for subsequent research.

**Phase II (2021–2023): Clinical adoption.** During this period, attribution methods were increasingly applied to clinical tasks involving MRI, CT, SPECT, and PET. Grad-CAM became the dominant approach because of its simple implementation and compatibility with existing CNN architectures. Representative studies explored Alzheimer’s disease classification using residual self-attention networks [19], Parkinsonian syndrome analysis using dopamine-transporter SPECT [21], and interactive prostate cancer diagnosis integrating Grad-CAM++ with textual clinical explanations [27].

This period reflects a transition from methodological development toward practical clinical applications.

**Phase III (2024–2026): Methodological diversification.** The most recent period is characterized by rapid growth and increasing methodological diversity. While Grad-CAM remains the most widely adopted attribution method, newer approaches address limitations in localization accuracy, anatomical interpretability, and explanation fidelity. Representative developments include atlas-guided LRP for brain age prediction [32], multi-scale prototypical explanations for glioma grading [30], anatomically guided attention mechanisms for glaucoma classification [35], and the introduction of generative Shapley attribution using diffusion bridge models [41].

These studies indicate a gradual shift from generic visualization techniques toward domain-specific and clinically grounded explanation strategies.

Overall, Grad-CAM remains the most frequently used method, while LRP, prototype-based approaches, and generative attribution techniques have gained attention in recent years.

### 4.2. Modality and Disease Coverage

Table 2 summarizes the imaging modalities, anatomical regions, and clinical applications represented in the reviewed literature. MRI/fMRI is the most frequently studied modality, accounting for 17 of the 32 included studies (53.1%), followed by CT with 7 studies (21.9%). SPECT and 3D OCT each account for 2 studies (6.3%), while CECT, PET, PET/MRI, and SPECT/CT are each represented by one study (3.1%). This distribution reflects both the widespread use of MRI in neurological disorders and the availability of publicly accessible volumetric neuroimaging datasets.

Accordingly, conclusions regarding method adoption and explanation quality are most strongly supported for MRI-dominated applications and should not be generalized without qualification to CT, PET, SPECT, OCT, multimodal imaging, or underrepresented anatomical regions and tasks.

The literature is concentrated on brain imaging and MRI-based classification, with limited coverage of thoracic, abdominal, cardiac, prostate, breast, retinal, multimodal, and segmentation applications. This imbalance restricts methodological development and leaves the generalizability of current 3D XAI methods across anatomical regions, imaging modalities, and clinical tasks insufficiently established.

### 4.3. XAI Method Distribution

We classified each study first by its general explanation approach and then by the specific methods applied or evaluated. The general categories included post-hoc attribution, intrinsically interpretable models, attention-based explanations, and generative explanations. Benchmarking was recorded as a study role rather than as a separate explanation method. A study could receive more than one method-family code when it applied or evaluated methods from multiple families.

Table 3 summarizes the distribution of XAI method families across the 32 selected studies. Grad-CAM and CAM variants were the most frequently reported family, occurring in 21 studies (65.6%). LRP was reported in five studies (15.6%), while Integrated Gradients or conventional saliency approaches occurred in four studies (12.5%). SHAP-based approaches were identified in two studies (6.3%). Prototypical networks and generative Shapley attribution were each represented by one study (3.1%). Because studies applying or evaluating methods from multiple families were counted in each applicable category, these frequencies are not mutually exclusive, and the total number of method-family occurrences exceeds 32.

### 4.4. The Dimensionality Gap

A central finding of this review is the mismatch between the dimensionality of model inference and explanation presentation. The included studies analyze volumetric medical images, most do not preserve 3D information throughout the complete explanation pipeline. Based on the dimensionality taxonomy introduced in Section 3, the reviewed studies are distributed as follows:

(15) $$\underset{\text{3D data processed as 2D}}{\underset{︸}{n_{P2}=2}}\;+\;\underset{\text{3D model with 2D visualization}}{\underset{︸}{n_{P3}=29}}\;+\;\underset{\text{3D inference, attribution, and visualization}}{\underset{︸}{n_{P4}=1}}\;=\;32,$$

Within the selected corpus of 32 studies, two were classified as P2, 29 as P3, and one as P4. The two P2 studies were retained as boundary cases illustrating how volumetric examinations can be processed as independent 2D slices; they do not represent an exhaustive sample of slice-based approaches. Among the 30 studies using volumetric model inference, 29 (96.7%) presented their explanations primarily through 2D views (P3), while one (3.3%) was classified as preserving 3D representation through visualization (P4).

The P2 cases differ in how they use activation maps. Hasan et al. [40] applied Grad-CAM and LIME to individual MRI slices and performed 3D reconstruction only after slice-based analysis. In contrast, Ali et al. [23] used class activation maps in a slice-based CT pipeline for training and augmentation rather than as a standalone explanation output. We retain the latter as a boundary case for dimensionality analysis, not as evidence of a clinically presented attribution map. The vast majority of studies fall within *P3*, where the DL model operates directly on the complete volumetric input, but the resulting attribution maps are presented as axial, coronal, or sagittal slice overlays. This includes most neuroimaging studies, brain tumor analysis, and nuclear medicine applications [34,38]. Attribution is computed over the entire volume, and the final explanation is reduced to a sequence of 2D visualizations, limiting interpretation of spatial continuity across anatomical structures.

P4 remains largely unexplored in the selected literature. Respond-CAM [9] generates a three-dimensional class-specific saliency map from convolutional feature maps and interpolates it to the input volume dimensions. The original study presents these results using projected three-dimensional contours or isosurfaces together with corresponding two-dimensional slices. Respond-CAM should be understood as a volumetric activation-mapping method, rather than as a native input-resolution voxel-wise attribution method.

These findings indicate that advances in 3D DL have progressed more rapidly than advances in volumetric explainability, highlighting native 3D attribution and visualization as important areas for further research.

### 4.5. Evaluation Landscape

Evaluation approaches were classified into two broad groups: qualitative evaluation and quantitative attribution-specific evaluation. Qualitative evaluation included visual inspection of attribution maps, comparison with known anatomical regions, and assessment by clinical or domain experts. Quantitative evaluation included faithfulness, localization, robustness, and related measures calculated specifically for the explanation rather than for predictive performance.

Among the 32 selected studies, four (12.5%) reported attribution-specific quantitative evaluation. The remaining 28 studies (87.5%) relied primarily on qualitative inspection, expert or anatomical comparison, or did not report a distinct evaluation of explanation quality. Classification accuracy, AUC, Dice score, and other predictive-performance measures were not counted as explanation-quality metrics unless they were used explicitly to evaluate the attribution.

The quantitative studies used different evaluation dimensions. SE3D [10] evaluated volumetric localization using VxAP, MaxF1, and Max3DBoxAcc. Chakraborty et al. [11] and Chrabaszcz et al. [12] evaluated faithfulness using perturbation-based measures, including AOPC and AUPC. Respond-CAM [9] evaluated its sum-to-score property using the $L_{1}$ error between the summed saliency-map values and the corresponding class score, together with Kendall’s τ for ranking agreement. It did not use pointing-game, deletion, or insertion metrics. Robustness and clinical-expert validation were recorded as separate evaluation categories when explicitly reported; they were not inferred from visual examples or predictive performance alone.

### 4.6. Thematic Synthesis of the Evidence

To consolidate related findings across the included studies, Table 4 organizes the results into four themes corresponding to method adoption, application coverage, explanation dimensionality, and evaluation practice. This thematic synthesis complements the study-level information in Table 1 by connecting the principal numerical findings to their methodological implications.

### 4.7. Overview of Included Studies

Table 1 summarizes the 32 selected studies by imaging modality, clinical task, explanation method or study role, dimensionality paradigm (P2–P4), and principal contribution or limitation. The two P2 boundary cases are identified separately so their role in the dimensionality analysis is clear.

As no formal risk-of-bias assessment tool was applied (Section 2), per-study risk-of-bias ratings are not reported. The “Key Finding/Limitation” column in Table 1 summarizes study-specific methodological limitations identified during data extraction and should not be interpreted as a formal risk-of-bias assessment. No included study received a formal critical-appraisal or risk-of-bias rating. Accordingly, the study-level findings reported in Table 1 are descriptive and were not weighted or filtered according to an appraisal score.

Collectively, the included studies illustrate the methodological evolution of XAI for 3D medical imaging, from early attribution approaches to more recent advances in volumetric explainability, quantitative evaluation, and clinically oriented applications. The table provides a consolidated reference for the comparative analyses presented in the following sections.

## 5. Discussion

This section interprets the principal findings of the review, with particular emphasis on the dominance of Grad-CAM, the role of LRP, the limited evaluation of volumetric explanations, and the need for native 3D explainability methods. Variability across the included studies is examined qualitatively in relation to XAI method family, imaging modality, and dimensionality paradigm, following the review framework described in Section 2.

The principal conclusions follow from the distributions reported in Section 4: Grad-CAM/CAM methods are the most frequent family in Table 3; the dimensionality analysis shows that 29 of the 30 studies using volumetric model inference were classified as P3; and the evaluation analysis identifies only four of the 32 selected studies as using attribution-specific quantitative measures. These results motivate the discussion below of spatial resolution, 3D visualization, and evaluation.

### 5.1. Grad-CAM Dominance and Resolution Limitations

Grad-CAM and its variants are the most frequently adopted attribution methods in the reviewed literature because of their compatibility with convolutional architectures and broad software support. Grad-CAM generates explanations from low-resolution feature maps in the final convolutional layer. Upsampling these maps to the input dimensions cannot recover spatial information lost during feature extraction (Equation (5)).

This limitation is particularly important in 3D medical imaging, where small lesions and fine anatomical structures may occupy only a few voxels. Consequently, Grad-CAM can provide clinically useful regional evidence but may produce coarse explanations that are insufficient for precise voxel-level localization.

Additional challenges arise when Grad-CAM is applied to segmentation models. Unlike classification networks, which produce a single class score, segmentation networks generate dense voxel-wise outputs. Adapting Grad-CAM to these outputs requires methodological modifications that remain only partially addressed in the current literature.

### 5.2. LRP in 3D Medical Imaging

LRP is the second most frequently adopted attribution method in the reviewed studies, with applications concentrated primarily in neuroimaging. By propagating relevance backward through the network, LRP can return relevance values to the input dimensions without upsampling a final convolutional feature map (Equation (9)).

The reviewed studies apply LRP to Alzheimer’s disease, multiple sclerosis, Parkinson’s disease, and brain-age prediction, where it identifies anatomically meaningful relevance patterns. Its use remains limited outside neuroimaging, leaving its effectiveness across CT, PET, SPECT, OCT, and other volumetric modalities insufficiently established.

### 5.3. Evaluation of 3D Attribution Methods

A major finding of this review is the limited use of quantitative evaluation for 3D attribution quality. Most studies rely on visual inspection or expert interpretation, while relatively few assess faithfulness, localization, or robustness using formal metrics. This lack of standardized evaluation limits comparisons across methods, datasets, imaging modalities, and clinical tasks.

Dedicated benchmarking frameworks such as SE3D represent an important step toward more objective assessment. Standardized protocols for volumetric explainability remain at an early stage of development. A further challenge is the definition of attribution ground truth. Lesion or anatomical masks identify clinically relevant regions but may not capture all image features influencing a model prediction. Localization metrics should be interpreted with caution and used alongside faithfulness-based measures rather than as the sole indicator of explanation quality.

The applicability of individual findings is also constrained by differences in imaging modalities, clinical tasks, datasets, and explanation-evaluation practices across studies. A visually plausible heatmap or a result from one dataset does not establish that an attribution method will remain faithful or clinically useful across scanners, institutions, or patient populations. Accordingly, the patterns identified in this review describe reported research practices rather than validated performance across clinical settings.

### 5.4. Toward Native Volumetric Explainability

The review reveals a persistent gap between volumetric model inference and explanation visualization. Many models process complete 3D images, their explanations are commonly presented through selected 2D slices.

Future methods should preserve spatial relationships across attribution generation and visualization while supporting clinically familiar views such as axial, coronal, and sagittal planes. Native 3D rendering should complement, rather than replace, slice-based interpretation, particularly where lesion extent and anatomical continuity are important.

The predominance of P3 studies, 29 of the 30 studies using volumetric model inference, suggests that clinicians are usually shown only selected views of a volumetric attribution. Such presentation may make lesion extent, inter-slice continuity, and relationships between separated anatomical regions more difficult to inspect. This review did not evaluate whether 2D presentation changes diagnostic accuracy or clinical decisions; this potential impact requires direct clinician-centered evaluation.

### 5.5. Patient Data Privacy and Clinical Use

Volumetric medical images can contain sensitive patient information, making data governance relevant when attribution maps or interactive explanations are stored, shared, or evaluated by clinicians. This review did not systematically extract privacy safeguards, consent procedures, or data-sharing policies from each included study. It cannot assess the adequacy of those safeguards across the corpus. Future evaluations should report the provenance and permitted use of imaging data and explain how patient information is protected when images and explanations are displayed or shared.

### 5.6. Limitations of the Included Studies and Review Process

The included studies vary in imaging modality, clinical task, dataset, and evaluation practice. Many present explanations through selected 2D views, and relatively few report attribution-specific quantitative evaluation. These differences limit direct comparison between methods and prevent the mapped findings from establishing clinical generalizability across scanners, institutions, or patient populations.

This review has several limitations arising from the methodology used. First, the literature search was conducted up to a single cutoff date (May 2026); studies published or indexed after this date are not captured, and the field of XAI for 3D medical imaging is evolving rapidly. Second, eligibility was restricted to English-language, peer-reviewed articles and publicly accessible preprints. Non-English studies and preprints on servers other than arXiv were not searched, and arXiv coverage was further limited to the cs.CV, cs.LG, and eess.IV categories. Relevant work indexed elsewhere or published in other languages may have been missed.

The PRISMA flow diagram reports 74 full-text exclusions under the broad reason “no 3D model,” while two studies using independent 2D slices from volumetric examinations were retained as P2 boundary cases. Because the excluded records were not reassessed under a separate P2-specific rule, the two P2 studies should not be interpreted as an exhaustive sample of slice-based approaches. Consequently, the distribution of dimensionality paradigms describes the selected corpus rather than their prevalence across the wider literature.

The available research log does not preserve the complete database-specific search strings, search fields, execution dates, retrieval counts, deduplication procedure, or record-level full-text exclusion decisions. These details cannot be reconstructed reliably from the selected studies, which limits independent replication of the search and screening process.

As discussed in Section 2, no formal risk-of-bias or critical-appraisal tool was applied to individual studies, limiting systematic comparison of their methodological quality. Grey literature and unpublished results were not systematically searched, and risk of bias due to missing results or selective reporting was not formally assessed. Studies reporting null or negative findings may be underrepresented. Finally, the review protocol was not prospectively registered, which may limit transparency regarding pre-specified versus post hoc analytic decisions.

## 6. Open Challenges and Research

The reviewed literature shows substantial progress in adapting XAI methods to volumetric medical imaging, but important limitations remain in attribution resolution, segmentation-specific explanations, multimodal interpretation, quantitative evaluation, and clinical validation. Addressing these challenges is necessary for developing explanations that are both technically reliable and clinically meaningful.

### 6.1. Resolution-Preserving Volumetric Attribution

The widespread adoption of Grad-CAM reflects its practical value for explaining deep learning models. Its reliance on low-resolution convolutional feature maps limits spatial precision. As discussed in Section 3, repeated pooling operations reduce the resolution of the attribution map before it is upsampled to the original volume size. This limitation is particularly important in volumetric medical imaging, where clinically relevant abnormalities may occupy only a small number of voxels.

Future research should investigate methods that preserve spatial detail throughout the attribution process. Promising directions include full-resolution gradient-based approaches such as Integrated Gradients [6], perturbation-based methods adapted to volumetric data, such as Score-CAM [45], and relevance propagation techniques designed for modern 3D architectures, including Swin UNETR [46] and nnU-Net [47]. Comparative studies across imaging modalities and clinical tasks should evaluate trade-offs among localization accuracy, faithfulness, robustness, and computational efficiency.

### 6.2. Segmentation-Specific Explainability

Most attribution methods were originally developed for classification and later applied to segmentation without substantial modification. Unlike classification models, segmentation networks produce dense voxel-wise outputs, requiring explanations to be conditioned on a selected region or predicted structure.

A segmentation-specific attribution formulation can be expressed as

(16) $$T_{c}^{\mathrm{seg}}\left(x;q\right)=\sum_{d,h,w}f_{c}{\left(x\right)}_{d,h,w}\;q_{d,h,w},$$

(17) $$A_{c}^{\mathrm{seg}}\left(x,q\right)=A\;\left(x;T_{c}^{\mathrm{seg}}\right),$$

Here, $T_{c}^{\mathrm{seg}}$ is the scalar target being explained, q specifies the output region of interest, and A(x;T) denotes an attribution method applied to input x with respect to scalar target *T*.

This formulation enables attribution to focus on a selected lesion, organ, or predicted segment rather than a global output score. Future work should develop and validate methods designed specifically for volumetric segmentation.

### 6.3. Multi-Modal Explainability

Clinical imaging increasingly combines complementary modalities such as PET/CT, PET/MRI, and multiparametric MRI. Multimodal models integrate information across these inputs, existing attribution methods rarely quantify the contribution of each modality.

A multimodal attribution decomposition may be expressed as

(18) $$S_{c}\left(x^{\left(1\right)},\ldots ,x^{\left(M\right)}\right)-S_{c}\left({\bar{x}}^{\left(1\right)},\ldots ,{\bar{x}}^{\left(M\right)}\right)=\sum_{m=1}^{M}C_{c}^{\left(m\right)}+I_{c},$$

where $C_{c}^{\left(m\right)}$ denotes the contribution assigned to modality *m*, $I_{c}$ represents residual cross-modal interaction effects, and $\left({\bar{x}}^{\left(1\right)},\ldots ,{\bar{x}}^{\left(M\right)}\right)$ is a reference input. This equation represents a desired attribution decomposition rather than a property guaranteed by every multimodal explanation method.

### 6.4. Standardized Benchmarking and Evaluation

Quantitative evaluation remains limited across the reviewed literature, with most studies relying primarily on visual assessment. Progress requires standardized datasets, evaluation protocols, and reporting practices that support consistent comparison across attribution methods.

Future benchmarks should include classification, regression, and segmentation tasks and assess multiple properties of explanation quality, including faithfulness, localization, robustness, computational efficiency, and reproducibility. Using complementary metrics is essential because no single measure fully captures the reliability of a volumetric explanation.

### 6.5. Clinical Translation and Foundation Models

Clinical deployment remains limited despite rapid methodological development. Future studies should evaluate attribution methods across diverse scanners, acquisition protocols, institutions, and patient populations, while examining their effects on clinician confidence, diagnostic performance, and workflow integration.

Large-scale pretrained models, including Segment Anything Model (SAM)-Med3D [48] and SuPreM [49], introduce additional explainability challenges because of their multi-scale representations and prompt-driven inference. Widely used frameworks such as nnU-Net further highlight the need for attribution methods that generalize across complex volumetric architectures. Developing scalable and clinically validated explanation techniques for these models remains an important research direction.

## 7. Conclusions

This scoping review presents a comprehensive analysis of explainable AI attribution methods for 3D medical imaging, synthesizing 32 primary studies published between 2018 and 2026. The review examined the evolution of attribution methods, imaging modalities, clinical applications, evaluation strategies, and explanation dimensionality through a unified analytical framework.

The reviewed literature shows that Grad-CAM remains the most widely adopted attribution method because of its broad applicability and ease of integration. LRP, IGs, SHAP, and emerging prototype- and generative-based approaches have been explored less frequently. The review identifies a persistent dimensionality gap, with most studies reducing volumetric explanations to 2D visualizations despite operating on inherently 3D medical images. Quantitative evaluation of attribution quality also remains limited, underscoring the need for standardized benchmarking and evaluation protocols.

The proposed dimensionality taxonomy (P2–P4) provides a structured framework for categorizing existing approaches according to explanation dimensionality. The unified mathematical framework establishes a common basis for comparing representative attribution methods and evaluation metrics across different medical imaging applications.

Future research should focus on developing resolution-preserving attribution methods, explainability techniques designed for volumetric segmentation and multimodal imaging, standardized quantitative benchmarks, and clinically validated evaluation frameworks. Progress in these areas will support the development of more reliable, interpretable, and clinically meaningful explainable AI systems for 3D medical imaging.

## Figures and Tables

**Table 1:** Study-level characteristics of the 32 studies selected for this scoping review. For each study, the table reports the explanation method or study role, imaging modality, clinical task, dimensionality paradigm, and principal finding or limitation. P2 denotes independent slice-based inference from a volumetric examination; P3 denotes volumetric inference with explanation presented through 2D views; and P4 denotes volumetric inference, attribution, and spatial 3D presentation.

Reference	Method	Modality	Task	Paradigm	Key Finding/Limitation
[9]	Respond-CAM	CECT	Classify	P4	Three-dimensional class-specific saliency map derived from convolutional features and interpolated to the input volume; evaluated using $L_{1}$ error and Kendall’s τ
[14]	LRP	MRI	Cls. (AD)	P3	LRP > guided backprop for hippocampal specificity
[15]	LRP	MRI	Cls. (MS)	P3	Highlights individual white matter lesions
[16]	CAM	OCT	Classify	P3	First 3D XAI on raw ONH OCT; Area Under Curve (AUC) =0.94
[17]	Grad-CAM	CT	Cls./Seg	P3	PyTorch (v2.11.0) library; 3D maps visualized as 2D slices only
[18]	SHAP	DCE-MRI	Regression	P3	Volumetric breast density estimation with SHAP-based model interpretation
[19]	3D Grad-CAM	MRI	Cls. (AD)	P3	Final-layer res. 6×7×6; first XAI for MCI
[20]	3D Grad-CAM	CT	Classify	P3	97.17% accuracy; $27^{3}$ nodule patches
[21]	LRP	SPECT	Cls. (PD)	P3	Data-driven extrastriatal discovery; 97.0% acc.
[22]	Saliency	PET	Cls. (DLB)	P3	Four-class dementia; putamen/thalamus identified
[23]	CAM	CT	Classify	P2	CAM used for training/augmentation rather than as a standalone clinical explanation; retained as a P2 boundary case
[24]	LRP	SPECT	Cls. (PD)	P3	Individualized maps improve physician trust
[25]	Saliency + CAM	fMRI	Cls. (SZ)	P3	+23% accuracy vs. magnitude CNN
[26]	Grad-CAM	CT	Risk pred.	P3	3D CNN for early recurrence prediction of hepatocellular carcinoma with spatial model interpretation
[27]	Grad-CAM++	MRI	Detection	P3	Interactive PI-RADS hybrid XAI; AUC =0.89
[28]	Grad-CAM fam.	fMRI	Cls. (AD)	P3	Five CAM variants; $14^{3}$ final-layer resolution
[29]	Grad-CAM/CAM	Dynamic Contrast-Enhanced (DCE)-MRI	Cls. (Breast CA)	P3	Explainable 3D CNN for pCR prediction from 3D DCE-MRI
[10]	Benchmark	MRI	Classify	P3	Volumetric attribution benchmark; explanations evaluated on 3D data but not presented as native volumetric renderings
[12]	Agg2Exp	CT	Segment	P3	Gradient > perturbation; AOPC/AUPC metrics
[30]	Prototypical	MRI	Classify	P3	Multi-scale 3D prototypical parts; BraTS2021
[31]	Grad-CAM	MRI	Risk pred.	P3	Multimodal explainable AI for identifying patients at risk of lethal ventricular arrhythmias
[32]	LRP	MRI	Regression	P3	Atlas-parcellated LRP; cerebellar importance
[33]	Grad-CAM	MRI	Cls. (PD)	P3	414 Julich atlas regions; n=200
[34]	Grad-CAM	MRI	Segment	P3	Grad-CAM not native to segmentation
[35]	CAREs+CAM	OCT	Classify	P3	Anatomically grounded; multi-task consistency
[36]	Grad-CAM	PET/MRI	Classify	P3	PET AUC =0.92; n=20 test set
[37]	Grad-CAM	MRI	Detection	P3	Grad-CAM applied to a 3D CNN for focal cortical dysplasia detection
[38]	Grad-CAM	MRI	Segment	P3	Explainable dual-modality framework for 3D brain tumor segmentation; explanations presented through 2D views
[39]	Grad-CAM	SPECT/CT	Prediction	P3	Explainable deep learning for predicting cardiac resynchronization therapy response
[40]	Grad-CAM/LIME	MRI	Classify	P2	“3D” title; DL inference on 2D slices
[11]	IG/CAM/Occ.	CT	Classify	P3	Cross-domain AOPC/AUPC/Compactness benchmark
[41]	S(H)NAP	CT	Risk pred.	P3	Diffusion Shapley; volumetric attribution for auditing Sybil bias

**Table 2:** Distribution of XAI method families across the 32 selected studies. Because each study was counted in every method family it applied or evaluated, the categories are not mutually exclusive, the total number of method-family occurrences exceeds 32, and the percentages do not sum to 100%.

Imaging Modality	Clinical Application	Representative Studies	*n*
CECT	Cellular tomography	[9]	1
MRI/fMRI (Brain)	Alzheimer’s disease	[14,19,28]	3
	Brain tumor/glioma	[10,30,34,38,40]	5
	Multiple sclerosis	[15]	1
	Parkinson’s disease	[33]	1
	Schizophrenia	[25]	1
	Brain age prediction	[32]	1
	Focal cortical dysplasia	[37]	1
MRI (Breast)	Breast density/pCR prediction	[18,29]	2
MRI (Prostate)	Prostate cancer	[27]	1
MRI (Cardiac)	Arrhythmia risk prediction	[31]	1
CT (Chest)	Lung nodule/lung cancer	[20,41]	2
	COVID-19	[11,17,23]	3
CT (Multi-organ)	Anatomical segmentation	[12]	1
CT (Liver)	Hepatocellular carcinoma	[26]	1
PET	Dementia (DLB/AD/MCI)	[22]	1
PET/MRI	Head and neck cancer	[36]	1
SPECT	Parkinsonian syndromes	[21,24]	2
SPECT/CT	Heart failure/CRT response	[39]	1
3D OCT	Glaucoma	[16,35]	2
Total primary included studies			32

**Table 3:** Distribution of XAI attribution methods across the 32 included studies. Because individual studies may apply multiple attribution techniques, method counts may exceed the number of unique studies, and percentages may not sum to 100%.

Method Family	*n*	%	Key Characteristics
Grad-CAM/CAM variants	21	65.6	Broad CNN support; requires suitable feature maps; resolution deficit (Equation (5))
LRP (α-β, $z^{+}$)	5	15.6	Input-dimensional relevance attribution; conservation depends on the propagation rule; predominantly applied to neuroimaging
Integrated Gradients/Saliency	4	12.5	Gradient-based attribution; Integrated Gradients requires evaluations along a path from a baseline, whereas conventional gradient saliency can be noisy
SHAP/DeepSHAP	2	6.3	Game-theoretic attribution; computationally expensive for high-dimensional volumetric inputs
Prototypical Networks	1	3.1	Intrinsically interpretable learning through multi-scale prototypes
Generative Shapley	1	3.1	Diffusion-based interventional attribution for volumetric imaging

**Table 4:** Thematic synthesis of the principal findings across the 32 selected studies. Method-family frequencies are not mutually exclusive because a study may apply or evaluate more than one explanation method.

Theme	Evidence from the Review	Interpretation
Method adoption	Grad-CAM/CAM variants occurred in 21 studies (65.6%); LRP occurred in five studies (15.6%).	The literature remains dominated by activation-mapping methods, despite their limited spatial resolution. LRP and other input-level methods have received substantially less attention.
Application coverage	MRI/fMRI accounted for 17 studies (53.1%), with substantial concentration in brain imaging and classification tasks.	The findings are most strongly supported for neuroimaging and should not be generalized without qualification to other modalities, anatomical regions, or clinical tasks.
Explanation dimensionality	Two studies were classified as P2, 29 as P3, and one as P4. Among the 30 studies using volumetric inference, 29 (96.7%) presented explanations primarily through 2D views.	Most explanation pipelines preserve volumetric information during inference but reduce it during presentation, limiting the inspection of spatial continuity and anatomical extent.
Evaluation practice	Four studies (12.5%) reported attribution-specific quantitative evaluation; 28 (87.5%) relied primarily on qualitative assessment or did not separately evaluate explanation quality.	Evidence regarding explanation faithfulness, localization, robustness, and clinical usefulness remains limited and difficult to compare across studies.

## Data Availability

The original contributions presented in this study are included in the article. Further inquiries can be directed to the corresponding author.
